# Escape from Human Immunodeficiency Virus Type 1 (HIV-1) Entry Inhibitors

**DOI:** 10.3390/v4123859

**Published:** 2012-12-19

**Authors:** Christopher J. De Feo, Carol D. Weiss

**Affiliations:** Office of Vaccine Research and Review, Center for Biologics Evaluation and Research, US Food and Drug Administration, 8800 Rockville Pike, Bethesda, MD 20892, USA; E-Mail: christopher.defeo@fda.hhs.gov

**Keywords:** resistance, entry inhibitor, virus entry, fusion, envelope glycoprotein, HIV

## Abstract

The human immunodeficiency virus (HIV) enters cells through a series of molecular interactions between the HIV envelope protein and cellular receptors, thus providing many opportunities to block infection. Entry inhibitors are currently being used in the clinic, and many more are under development. Unfortunately, as is the case for other classes of antiretroviral drugs that target later steps in the viral life cycle, HIV can become resistant to entry inhibitors. In contrast to inhibitors that block viral enzymes in intracellular compartments, entry inhibitors interfere with the function of the highly variable envelope glycoprotein as it continuously adapts to changing immune pressure and available target cells in the extracellular environment. Consequently, pathways and mechanisms of resistance for entry inhibitors are varied and often involve mutations across the envelope gene. This review provides a broad overview of entry inhibitor resistance mechanisms that inform our understanding of HIV entry and the design of new inhibitors and vaccines.

## 1. Introduction

Since zidovudine (AZT) was approved in 1987 as the first drug for treating human immunodeficiency virus (HIV) infection, more than twenty drugs representing antiretroviral classes that inhibit five different steps in the viral lifecycle have reached the clinic [1,2]. For those with access to these drugs and medical care, this unprecedented progress in developing agents to treat a viral infection has led to the transformation of HIV infection from a progressively fatal disease to a chronic, manageable infection [3,4]. Efforts to develop more drugs continue because the high replication rate of the virus and high error rate of the HIV polymerase [5,6] generate a continuous supply of mutations that facilitates development of drug resistance. 

While each step of the viral lifecycle affords an opportunity for therapeutic intervention, most of the approved antiretroviral drugs inhibit three viral enzymes—reverse transcriptase, integrase, and protease—which respectively catalyze the replication and integration of the viral genome in the host cell and the maturation of the viral core to form new viral particles. These enzymes function in the homeostatic intracellular environment, and, consequently, their respective genes have relatively little sequence diversity [2]. The two other classes of approved antiretroviral drugs target the viral envelope glycoprotein (Env) and inhibit virus entry by interfering with Env binding to the chemokine coreceptor or Env mediating fusion between the virus and host cell membranes [7,8,9,10]. The binding and fusion activities of Env typically take place in extracellular environments where Env negotiates heterogeneous cellular targets under continuously evolving antibody pressure. Consequently the *env* gene has relatively high sequence diversity [2]. The myriad of factors influencing the function of these viral proteins affect how resistance evolves to an inhibitor. 

**Figure 1:**
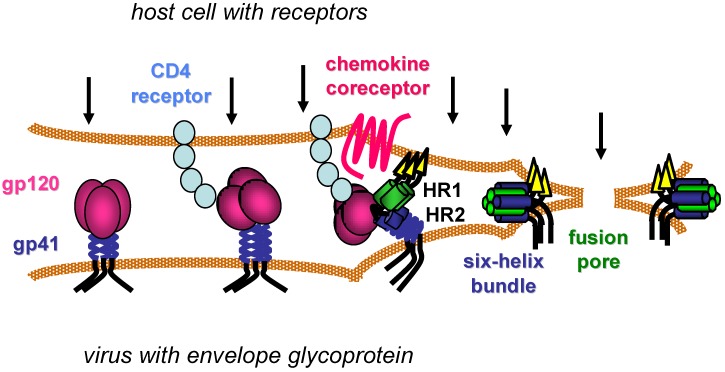
Model of HIV entry. CD4 receptors and chemokine coreceptors are shown on the host cell. The gp120 surface subunit and gp41 transmembrane subunit of the HIV envelope glycoprotein are shown on viral membrane (envelope). After gp120 binds to CD4, the envelope glycoprotein undergoes conformational changes that facilitate gp120 interaction with the chemokine co-receptor. Additional conformational changes in the gp41 transmembrane subunit transiently expose two heptad-repeat domains (HR1 and HR2) that subsequently self-assemble to form a six-helix bundle structure. Formation of several gp41 six-helix bundles bring the host and viral membranes together for fusion, while several six‑helix bundles likely coalesce to form a fusion pore that allows the viral core to pass into the host cell cytoplasm. Arrows indicate potential steps in the entry process for inhibition.

HIV begins its life cycle when Env attaches to target cells (Figure 1), often first in a non-specific manner [11], before engaging the CD4 cell surface receptor on one of several types of CD4^+^ immune cells [12,13,14,15]. CD4 is the first of two receptors required for HIV infection. Specific interactions between Env and CD4 then induce conformational changes in the trimeric Env complex, which include exposure of new epitopes in the gp120 surface subunit and still undefined changes in non‑covalent interactions between gp120 and the gp41 transmembrane subunit. These conformational changes facilitate binding of gp120 to a chemokine coreceptor, either CXCR4 or CCR5 depending on the Env sequence [16,17,18,19,20,21]. Oligomerization, post-translational modifications, cell surface localization and expression levels of the chemokine and CD4 receptors vary with cell types and contexts, and such features affect productive interactions with Env [22,23].

Coordinated engagement of CD4 and the chemokine receptor at the host cell surface activates the membrane fusion activity of the gp41 transmembrane subunit, which is believed to involve repositioning of the hydrophobic N-terminus of gp41 (fusion peptide) to allow its insertion into the host cell membrane. This movement exposes two heptad-repeat regions (HR1 and HR2) in the gp41 ectodomain that subsequently self assemble into a thermostable six-helix bundle (6HB) structure. Three HR1 domains from each monomer of the Env trimer form a triple-stranded, coiled-coil core, against which three HR2 helices pack in its grooves in an antiparallel manner. Formation of the 6HB conformation provides a critical driving force that brings the viral and host cell membranes together, facilitating membrane merger and ultimately formation of an expanding fusion pore that allows the viral core to pass into the host cell cytoplasm [24,25].

This review summarizes concepts in the emergence of resistance to entry inhibitors. The entry inhibitors that will be discussed cover the major steps in HIV entry: agents that interrupt productive interactions between Env and the CD4 receptor or between Env and the chemokine co-receptor, agents that interfere with Env-mediated fusion between virus and host cell membranes, and other inhibitors that are not easily classified. The discussion selects examples of inhibitors for which *in vitro* or *in vivo* resistance data are available to highlight particular points, but makes no attempt to include all entry inhibitors in the published literature [7,8,9,10]. The perspective centers on insights into the mechanism of virus entry rather than on the practical application of therapeutics in the clinic. The ideas conveyed will nonetheless hopefully form the basis for new strategies for developing and using entry inhibitors.

## 2. Inhibitors of Envelope Glycoprotein-CD4 Receptor Interactions

### 2.1. Introduction

**Figure 2:**
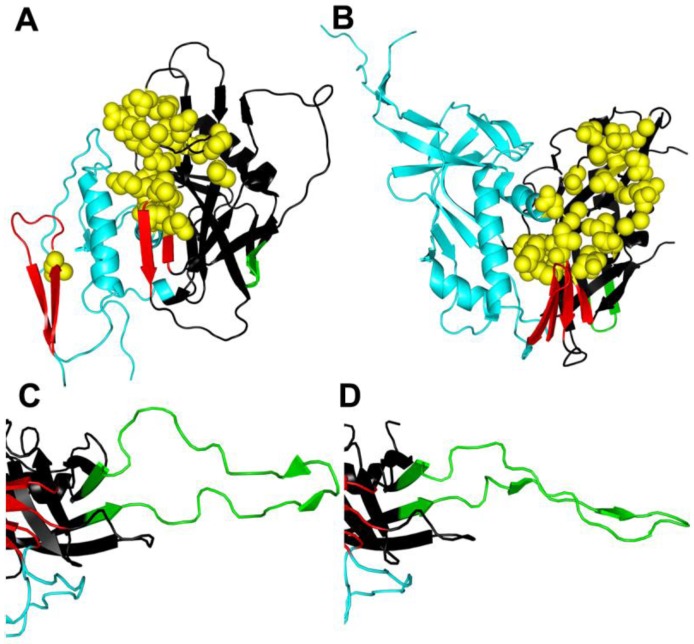
Conformational changes in gp120. (**A**) Unliganded simian immunodeficiency virus (SIV) gp120 core [32]. (**B**) HIV gp120 core complexed with CD4/48d antigen-binding fragment (Fab) [40] reveal significant conformational changes when CD4 is bound. (**C**) V3 in CD4/X5 Fab gp120 core complex [41]. (**D**) V3 in CD4**/**tyrosine-sulfated 412d Fab gp120 core complex [42] reveals potential conformational changes that can occur when V3 binds to CCR5. Resistance mutations to entry inhibitors often occur in the CD4 binding site (yellow spheres) and in the V3 domain (green), but can also be found elsewhere in gp120 and gp41. Resistance mutations may alter conformational changes in Env in ways that go beyond the classical resistance mechanism of excluding the inhibitor from its binding site. The inner domain is colored cyan, the outer domain is black, the bridging sheet is red, the V3 loop (or stem in (**A**) and (**B**)) is green, and residues contacting CD4 are represented as spheres and colored yellow. The PDB accession codes that used were 2BF1 (**A**), 3JWD (**B**), 2B4C (**C**), and 2QAD (**D**). For clarity only gp120 is displayed.

The identification of CD4 as the initial receptor for HIV created opportunities for developing inhibitors, but no candidates have been approved for clinical use to date. CD4 binds to a depression in gp120 that is formed by the intersection of its inner and outer domains, and a 4-stranded β-sheet that is important for coreceptor binding [26] (Figure 2A,B). Biochemical, biophysical, and structural studies indicate that CD4 can induce large conformational changes in gp120 between the native and CD4‑bound states [27,28,29,30,31,32,33,34]. However, recent structural work suggests that gp120 may have evolved to “snap” into the CD4-bound conformation, which may be the energetically-favored ground state that CD4 stabilizes [35]. Prior to receptor binding, gp120 variable loops and interactions with gp41 likely restrict this transition to lower energy conformations [35]. Such restricting interactions vary among different Envs, supporting the possibility of many unliganded conformations of gp120 [35,36,37,38,39]. This structural and functional heterogeneity presents challenges for developing inhibitors that interrupt activation of Env by CD4. Nevertheless, some broadly potent candidates have been developed, and Env adapts to them in various ways to gain resistance. 

### 2.2. Protein Inhibitors of Env-Receptor Interactions

An early candidate, soluble CD4 (sCD4), which can bind to Env tightly, is able to potently inhibit Env and block virus entry, but in some cases it can also activate Env and enhance entry [43,44,45,46,47,48,49,50,51]. Furthermore, while effective against most lab-adapted viruses, sCD4 performs poorly against many primary viruses [52,53,54,55,56,57]. Interestingly, differences in sCD4 potency often did not correlate with sCD4 affinity for soluble gp120, suggesting that sCD4 does not solely inhibit by a competitive mechanism [53,54,55,56]. An additional mechanism of inhibition proposed that sCD4 causes irreversible shedding of gp120 from gp41, thereby inactivating Env, but this again was noted primarily for lab‑adapted viruses and still does not always explain inactivation at all concentrations of sCD4 [27,54,56,57,58,59]. This indicated that other mechanisms were involved [56]. In the end, the difficulties in understanding sCD4 inhibition were compounded by poor performance in the clinic. 

Despite these early setbacks, efforts to identify therapeutics that target this key step in the entry process continue. A series of CD4-mimetic miniproteins, which are easier to synthesize and chemically manipulate for improved binding compared to sCD4, were rationally designed using an unrelated protein scaffold containing a CDR2-like loop that is structurally homologous to the region of CD4 that binds gp120 [60,61,62]. The CD4M33 miniprotein, for example, has residues on the loop of the scorpion toxin scyllatoxin that were modified to conform to residues of CD4. After binding to gp120, CD4M33 induces conformational changes in Env that are similar to those induced by sCD4 and inhibits HIV in the low micromolar to nanomolar range [61]. The smaller size of the mimetics compared to sCD4 may allow better access to the CD4bs that may be partially occluded by variable loops in some strains, especially in primary viruses. 

Resistance studies with sCD4 and CD4 mimetics have consistently revealed mutations in the expected regions involved in gp120-CD4 interactions. High-level resistance readily emerged against both sCD4 and CD4-mimetic miniproteins [63,64]. Resistance mutations tended to replace highly‑conserved residues that are part of or close to the CD4bs (e.g., H105, V225, S375, G471), although less conserved mutations were noted as well [63]. Similar mutations confer resistance to sCD4 and human neutralizing sera containing antibodies to the CD4bs [64,65,66]. Although many of these mutated residues do not appear to make direct contact with the inhibitor, they clearly could alter interactions within the CD4 binding cavity. In fact, some mutations did reduce the binding affinity of gp120 for sCD4 and the mimetic, but the affinities did not always appear to correlate with the degree of resistance, suggesting that other resistance mechanisms were at work (see section below). Interestingly, viruses that were resistant to M48 mimetics also exhibited cross resistance to a variety of CD4bs inhibitors to some extent, and, in some cases, to V3-directed antibodies and neutralizing antibodies to CD4-induced (CD4i) epitopes. In all cases, viruses resistant to CD4bs inhibitors retained CD4 dependence for entry [63]. These findings suggest that resistance mutations can interfere with inhibitor binding (Table 1, mechanism A (I)) or possible induction of the coreceptor binding site through an allosteric mechanism (Table 1, similar to mechanism B (I)). Altogether, despite large binding footprints for many of these inhibitors, the complex network of interactions within the Env trimer affords several possibilities for escape. 

### 2.3. Small Molecule Inhibitors of Env-Receptor Interactions

While protein-based CD4bs inhibitors have the advantage of potency resulting from protein-protein interactions with Env, they lack the advantage of oral availability that is characteristic of small molecule inhibitors. Several groups have made progress in identifying small-molecule inhibitors of Env-CD4 interactions. One such class of inhibitors, N-phenyl-N-piperidin-4-yl-oxalamide (NBD) analogues, inhibits cell-cell and virus-cell fusion in the low micromole range [67]. Thus far, the lead compounds are not ready for the clinic, but they have been used successfully as tools to investigate gp120 interaction with CD4. An interesting feature of these small molecules is that they bind to gp120 with only low micromolar affinity, yet can induce large conformational changes in gp120 similar to those observed by sCD4—a remarkable accomplishment for any small molecule [68,69,70,71]. Additionally, the binding site has been identified in a recent crystal structure and is localized deep within a conserved hydrophobic cavity on gp120 called the phenylalanine 43 (Phe43) cavity, where the phenylalanine at position 43 of CD4 makes important conserved contacts with gp120 [26,35]. Recent work demonstrated that NBD analogues, as well as sCD4, can inactivate HIV-1 by prematurely triggering Env to an active, but metastable conformation that decays to an inactive form [72]. The lifetime of this intermediate varies for different Envs, even for Envs with similar affinities for CD4, possibly explaining differences in susceptibility to sCD4 and other inhibitors that target the CD4bs. 

Resistance mutations selected by NBD-556 are similar to those selected by sCD4 and CD4-protein mimetics, and again were concentrated around the Phe43 cavity [64]. However, as with the CD4 protein mimetics, not all mutations are predicted to involve residues that make direct contact with the inhibitor. One frequently emerging mutation from sCD4 and NBD-556 selections is a S375N substitution. This mutation as well as A433T and V255E are sufficient for NBD-556 resistance. Furthermore, these mutations also confer reduced susceptibility to a CD4-induced (CD4i) monoclonal antibody in the presence or absence of bound NBD-556 [64]. Interestingly, various mutations at position 375 influence conformational triggering and can either reduce or enhance NBD-556 binding, depending on the size of the amino acid [64,69]. An S375W mutation, for example, stabilizes the post‑CD4 binding conformation and reduces NBD-556 binding. Therefore, it appears that Env has the capability to escape NBD-556 inhibition by either preventing inhibitor binding (Table 1, mechanism A (I)) or adjusting how Env transitions to an activated state. In the later case, resistance mutations might be slowing the rate of NBD-induced inactivation (Table 1, mechanism B (I)). Additional studies are needed to confirm and refine these resistance mechanisms. 

Another class of small molecules that block virus entry, the BMS (Bristol-Myers Squibb) compounds, targets a similar region in gp120 as the NBD-556 analogs, but works by a different mechanism [73,74,75,76,77]. Initially it appeared that these molecules competitively block Env interactions with CD4 [73,77], but follow up experiments using viruses that do not require CD4 for entry were still inhibited in cells lacking CD4 receptors [78]. Rather, it was proposed that BMS compounds block events following CD4 interaction by preventing conformational changes that lead to HR1 exposure [78]. Additional experiments demonstrated that sCD4 and the BMS compounds can block each other’s binding, but this competition gets less efficient at higher sCD4 concentrations [79]. Also, in contrast to NBD-556 analogs, BMS compounds do not appear to induce a large enthalpically-driven conformational change like sCD4, although modest changes are detectable [68,79]. Furthermore, Env deletions in the gp41 cytoplasmic tail or V1V2 regions have strain-specific effects on inhibition by BMS drugs, such as accommodating both the drug and sCD4 [79]. Altogether, it seems that BMS compounds most likely interfere with both CD4 binding and conformational changes after CD4 binding.

Resistance studies involving various BMS inhibitors and the X4 viruses, NL4-3 and LAI, revealed mutations throughout Env, including two gp41 mutations found in two separate *in vitro* screens (I595F and K655E) [73,80]. Not surprisingly, mutations in gp120 were focused within or adjacent to the CD4bs, including but not limited to substitutions at residues M475, M434, M426 and F423. *In vivo* data for BMS resistance are also available. Of the eight patients who developed resistance, only three had mutations that were recovered in the *in vitro* screens (V68A and M426L), highlighting how the strain (Env context) and environment (*in vitro versus in vivo*) can affect resistance [76]. Additionally, the most prevalent resistance mutation *in vivo* was S375I/N, the same position that confers resistance to NBD molecules.

The mechanism for resistance to BMS molecules has not been completely defined. However, mutations such as M426L and M475I reduced susceptibility to BMS-378806 and significantly reduced binding of the inhibitor, suggesting that a competitive mechanism contributes at least partially to resistance [77] (Table 1, mechanism A (I)). Furthermore, more distant mutations (e.g., V68A) [73,80] enhanced resistance when combined with the CD4bs mutations, underscoring the long-range communication in Env used to modulate resistance [73,77]. Indeed, some of these resistance mutations also conferred increased sensitivity to HIV-infected sera and broadly neutralizing antibodies directed to the membrane external region (MPER) of gp41, suggesting that they cause global changes in Env [80]. Accordingly, because BMS prevents conformational changes necessary for fusion, the resistance mutations might counter BMS by making it easier to undergo those conformational changes (Table 1, mechanism B or C (I)), which is the opposite of what the resistance mutations appear to do for NBD analogs. 

Another class of small molecules being developed is the cyclotriazadisulfonamides (CADAs) that take aim at CD4, a cellular target, rather than the highly variable Env protein [81,82]. These molecules have broad HIV antiviral activity by knocking down CD4 expression (up to 90% of initial levels) in an mRNA-independent manner [81,83]. CD4 levels can become limiting for HIV infection, though corresponding levels of coreceptor are also important [84]. Despite CADA’s potency, knocking down CD4 levels in T cells may have detrimental long-terms effects on immune function and may pressure the virus to use CD4 more efficiently or switch tropism. 

A resistance study performed with CADA and the NL4-3 strain identified mutations that enhanced sCD4 sensitivity, but still required CD4 for entry [85]. Of note, these mutations, like those identified in other resistance screens, are spread throughout the gp120 molecule and include identical mutations obtained in the BMS resistance screens and BMS-treated patients (V68A and S440R) [73,76,80]. Despite the overlap of mutations identified between these different classes of small molecules, it is hard to predict the exact resistance mechanism to CADA. It likely involves more efficient use of CD4 (Table 1, mechanism C (1)) because CD4 levels are reduced in the presence of this drug [81,83,85], and resistance mutations confer increased sensitivity to sCD4 [85]. Such mutations may be analogous to those seen with lab adaption of primary viruses on cells with low levels of CD4. Regardless, even though CADA targets a cellular protein, mutations in Env can still confer resistance to CADA. Although selection for more efficient receptor use could be a drawback for therapy, this class of entry inhibitors may still have an important preventive role in microbicide applications [86].

### 2.4. Antibodies Targeting the CD4 Receptor

While this review does not cover antibodies that inhibit HIV entry, we are including this section on the monoclonal antibody (mAb), Hu5A8 (TNX-355; Ibalizumab), because its resistance profile has similarities with some of the other entry inhibitors covered in this review. Developing antibodies aimed at CD4 instead of Env could be a useful strategy, but such therapeutics would need to avoid interfering with the normal biological function of CD4. Ibalizumab binds to domain 2 of CD4, a location that interferes with CD4-induced activation of Env, but not normal CD4 T-cell immune function [87,88,89,90,91]. Although it does not prevent Env from binding to CD4, it blocks CD4-induced conformational changes [88,89]. How it does this is still not resolved. Early studies indicated that bivalency of the mAb may be important, but it was not essential for inhibiting cell-free virus entry, and some Envs were still sensitive to the Fab fragments alone [88,89,92]. Other studies looking at the structural properties of CD4 and its interaction with gp120 or ibalizumab did not provide any clarity on the mechanism of inhibition [92]. 

The potency of ibalizumab and its favorable performance in pre-clinical studies encouraged initiation of clinical trials, but resistance was still a factor, even though the mAb targeted a host antigen [87,90,91,93,94,95,96]. Analysis of clinical samples demonstrated that resistance reduces the maximum level of inhibition to roughly 33%–83% of wild type [96]. This finding suggests that these resistant Envs are able to side-step saturating levels of mAb bound to CD4 using non-competitive mechanisms, similar to the mechanism seen for resistance to CCR5 inhibitors (discussed in next section) [94,96]. Further, viruses resistant to ibalizumab also exhibited increased infectivity, increased sCD4 sensitivity, and decreased sensitivity to an antibody that competes with gp120 for CD4 binding (RPA-T4), though these viruses were not more sensitive to other broadly neutralizing antibodies or other fusion inhibitors. Sequence analysis revealed numerous mutations across Env, although none directly within the CD4bs. Despite these varied phenotypes, loss of V5 gylcosylation was identified as a major determinant for resistance. Most likely, this feature and other mutations work together to enhance CD4-induced conformational changes that overcome blocks created by the bound antibody (Table 1, mechanism B (I)).

### 2.5. Summary

Diverse inhibitors targeting Env-CD4 interactions frequently select for shared and unique resistance mutations that cluster in the CD4 binding cavity. These mutations often occur with other isolated mutations spanning distant regions of Env. The escape pathways depend on whether the mechanism of inhibition involves preventing or prematurely activating Env conformational changes needed for fusion. Subtle differences in how different inhibitors (e.g., BMS *versus* NBD analogs) bind to Env can result in different modes of inhibition. Resistance mutations in and around the CD4bs may directly or indirectly interfere with inhibitor binding (Table 1, mechanism A (I)) or they may alter how Env undergoes conformational changes to preserve Env function, whether or not the inhibitor remains bound to its target (Table 1, mechanism B (I)). The variety of mutations in Env that can be used to overcome these blocks underscores the built-in capacity of Env to alter the way it binds and responds to CD4. Similarly, recent structural work describes how sCD4 and the neutralizing mAbs, VRC01 and b12, which all bind to Env with overlapping footprints, can support distinct structural conformations upon binding [97]. Therefore, agents that block CD4 binding and activation must navigate the intrinsic dynamics of Env conformational changes required for entry. Resistance to these agents offers insights into the networks within Env that undergo reprogramming to satisfy viral entry. 

## 3. Inhibitors of Chemokine Receptor Interactions

### 3.1. Introduction

After gp120 binds to the CD4 receptor, the next therapeutic target in HIV entry is gp120 engagement with the coreceptor, an essential step in controlling conformational changes in Env that lead to membrane fusion [24,25,98]. Early studies identified two classes of viruses that were characterized by their ability to infect CD4^+^ T cells (T tropic) or macrophages (M tropic). Later it was discovered that T-tropic viruses use the CXCR4 chemokine receptor (called X4 viruses) as their coreceptor [16], while M-tropic viruses use the CCR5 chemokine receptor (called R5 viruses) as their coreceptor [17,18,19,20,21,99]. As happened with the identification of CD4, the identification of the coreceptors initiated intense efforts to design inhibitors to block Env engagement with the coreceptor. In fact, the inhibitory activity of natural chemokine ligands for CCR5 helped lead the way to the discovery that CCR5 was the coreceptor for M-tropic viruses [100]. Shortly after, SDF-α, the natural ligand for CXCR4, was also shown to inhibit entry of X4 viruses [101,102]. These examples provided a proof of principle for entry inhibitors directly targeting Env interactions with the coreceptors. 

CD4-induced changes in Env generally enhance exposure of V3 and both formation and exposure of the gp120 bridging sheet (Figure 2B), two determinants important for binding to the G-protein-coupled, chemokine receptors [26,28,29,30,32,41,42,103,104]. In most cases, the tip of V3 interacts with the chemokine receptor ECL2, while the stem and base of the V3, including the conserved bridging sheet, binds to the chemokine receptor N-terminus [41,42,105,106,107,108,109,110,111]. For R5 viruses, CCR5 engagement leads to further conformational changes in the extended V3 loop structure that accommodate critical sulfonated tyrosines in the N-terminus of CCR5 (Figure 2C,D) [106,112]. These residues bind to a pocket in the V3 base where they help to stabilize a rigid beta-hairpin structure [41,42,112]. 

Inhibition of HIV entry by chemokine analogues can involve partial agonist activity that leads to down regulation of the receptor, or it can involve direct receptor blockade [113,114,115,116,117,118]. By contrast, small molecule CCR5 inhibitors are allosteric inhibitors that alter the conformation of the receptor so that it can no longer be recognized by gp120, as well as some chemokines and mAbs [119,120,121,122,123,124,125,126,127,128,129,130,131,132,133,134,135]. They typically bind to a hydrophobic pocket formed by transmembrane helices of the chemokine receptor [120,123,130,131,135,136]. Many of these small molecule inhibitors, including the CCR5 inhibitors aplaviroc (APL), vicriviroc (VCV), and maraviroc (MVC), have advanced into the clinic [137]. However, despite success in identifying many inhibitors, only one compound targeting interactions with a coreceptor (MVC) is currently approved for clinical use by the FDA. Nevertheless, many *in vivo* and *in vitro* resistance studies involving various coreceptor inhibitors offer insights into the complex network of interactions that cooperate to maintain Env function. 

### 3.2. CXCR4 Inhibitors

Early *in vitro* resistance studies were performed with CXCR4 inhibitors and included peptides (e.g., T134) [138], the chemokine SDF-1α [139], and the bicyclams, a small-molecule class of inhibitors that includes AMD3100 [140,141]. Here the initial patterns of resistance became evident: mutations in V3 were most common, and other mutations in gp120 were consistently present, though their contributions to resistance were less clear. Cross resistance to other CXCR4 inhibitors, albeit to different levels, was also a common property of these resistance viruses, indicating similar resistance mechanisms. Interestingly, resistance to the polyanionic inhibitor dextran sulfate, which appears to bind to V3 and other conserved coreceptor binding sites on gp120, selected for some of the same resistance mutations in the V3 that were later identified for bicyclams [140,142,143]. 

Early investigations into the possibility that a selective chemokine receptor inhibitor might alter tropism gave mixed results. Resistance studies with the chemokine SDF-1α [139] or peptide T134 [138] and the X4 virus, NL4-3, did not select for viruses with CCR5 tropism. However, a different study, involving peripheral blood mononuclear cells infected with clinical isolates containing X4 and X4R5 viruses under AMD3100 selection, showed outgrowth of R5 viruses [144]. Therefore, studies with CXCR4 inhibitors indicated that a tropism switch is possible, but outgrowth of viruses that maintain CXCR4 tropism as a result of resistance mutations in gp120 are also likely to occur. 

### 3.3. CCR5 Inhibitors

#### 3.3.1. Tropism Switch

The idea that resistance to CCR5 inhibitors might lead to a coreceptor switch raised concerns that selection of X4 viruses could enhance disease progression. Emergence of X4 viruses correlates with worsening immunodeficiency, though it remains unclear whether they are its cause or consequence [145]. In any case, a switch to X4 viruses was not seen in early *in vitro* resistance studies with the chemokine MIP-1α using the JR_FL_ clone or with the CCR5 blocking mAb 2D7 using the JR_CSF_ clone and a primary isolate 11-121 [146,147]. Yet, when a resistance study was performed with derivatives of the CCR5 ligand RANTES, by infecting a human peripheral blood lymphocyte-SCID mouse model with the R5 242 isolate that only requires few changes to become dual or X4 tropic, a tropism switch was detected in the resistant viruses [148]. These conflicting results, therefore, did not provide early resolution on the likelihood of a tropism switch resulting from chemokine inhibitor use in the clinical setting.

As resistance studies began to focus on small-molecule inhibitors of CCR5, it became more apparent that selection of viruses that use CXCR4 is an uncommon mechanism of escape, at least *in vitro*. For studies with VCV (previously called SCH-D/SCH 417690), AD101 (a preclinical precursor of VCV), and MVC (previously called UK-427,857), which all used the R5 clinical isolate CC1/85 from a patient who eventually developed viruses that could use CXCR4, the majority of recovered resistant viruses maintained CCR5 tropism [127,149,150,151,152]. Similarly, in resistance studies using the R5 clade G isolate RU570 under VCV or MVC selection, or the isolate KK under TAK-652 (successor to the small molecule CCR5 inhibitor TAK779) selection, there again was little evidence for outgrowth of X4 or dual tropic viruses [119,126,151,153,154]. It was also noted that viruses in the VCV screen could replicate in U87-CD4-CXCR4 cells, but they most likely represented minor variants in the quasispecies [150]. Additionally, using the SF162 isolate under MVC selection, X4 viruses could be detected, but such viruses were also recovered in cultures passaged without MVC, suggesting that the tropism switch seen in the resistance cultures may have involved adaptation to the cell culture [151]. Therefore, irrespective of the starting isolate or CCR5 inhibitor, it appeared that a switch to CXCR4 tropism was not the favored pathway for resistance to CCR5 inhibitors *in vitro*. 

By contrast, treatment failure in the clinic involving APL, VCV, and MVC more frequently involved outgrowth of pre-existing X4 or dual-tropic viruses that were initially present as a minority population within the viral quasispecies [155,156,157,158,159,160,161,162,163,164]. In clinical trials involving the addition of MVC to a antiretroviral regimen in subjects who had incomplete viral suppression (MOTIVATE trials), X4 or dual-tropic viruses were detected in about 50% of subjects who failed regimens containing MVC compared with about 6% of control subjects who failed similar regimens lacking MVC [161,165]. Similarly, in a trial involving MVC-containing combination therapy in treatment-naïve subjects who had only R5 viruses at screening (MERIT trial), X4 viruses emerged in almost a third of MVC-treated subjects, but in none of the subjects whose regimen did not include MVC [166]. An additional report describes a remarkable case of an X4 MVC-escape variant resulting from recombination between a long-term, archived proviral sequence from an X4 virus and an R5 dominant strain circulating at the time of MVC treatment initiation [167]. Thus outgrowth of pre-existing, minority X4 viruses is a frequent, but not universal, outcome of patients treated with APL, VCV, and MVC (Table 1, mechanism C (II)). Moreover, outgrowth of X4 viruses might also be a less likely resistance pathway for certain subtypes, such as subtype C, in which X4 viruses are much less common [168,169].

#### 3.3.2. Altered CCR5 Use

R5 viruses isolated from *in vitro* resistance studies often replicated efficiently in the presence of the CCR5 inhibitor, and in some cases replication efficiency increased in the presence of a large excess of inhibitor [110,149,150,151,153,154,170,171,172,173,174]. These resistant viruses showed mutations throughout Env, although the majority were found in the V3 region [149,151,153,154,170,173,175,176]. Often multiple V3 mutations were necessary for full resistance, but deletions in the V3 could also confer resistance [110,151,154,170,174,175,176,177,178,179]. In some cases, similar mutations were identified when different CCR5 inhibitors and isolates were used, but more often the V3 resistance patterns were distinct. 

Similarly, R5 viruses isolated from patients treated with APL, VCV, and MVC had resistance mutations spread throughout Env, including within the V3 region, but the V3 mutations frequently contributed the most to resistance [133,155,156,163,180,181,182,183,184]. Sequence comparisons of recovered viruses from different patients showed a great deal of variation and little overlap with *in vitro* studies. For example, replacing histidine with proline at position 308 (H308P) in the V3 was shown to be important for conferring resistance to AD101 *in vitro*, but mutating a proline or threonine to a histidine (P/T308H) was important for conferring resistance to viruses derived from a patient treated with MVC [133,170]. On the other hand, K305R was seen in multiple *in vitro* studies, as well as in VCV‑treated patients [153,163,170,175,176]. Despite the variable pathways to resistance, viruses recovered from *in vitro* screens and patients often conferred cross resistance to different CCR5 inhibitors. Thus, the V3 region is clearly a critical determinant for resistance, but different genetic pathways can be used depending on the inhibitor and virus strain.

Regions outside the V3 can also play a primary role in resistance. During an *in vitro* VCV selection, two resistant viruses had no additional mutations in the V3, though they were still cross resistant to chemokine agonists and other small-molecule, CCR5 entry inhibitors [150]. The lack of any V3 mutations is even more remarkable since these resistant viruses were isolated from separate, but related parental isolates used in a previous AD101 resistance screen, which did select for V3 mutations [170]. Unexpectedly, mutations in the fusion peptide (FP) region of gp41 were shown to be substantially responsible for VCV resistance to these resistant viruses [172,185]. Furthermore, one of these FP-dependent resistant viruses was found to be less sensitive to anti-V3 mAbs and CD4 inhibitors compared to a resistant virus that took the V3 pathway to resistance [173]. These findings show that different genetic pathways for resistance to CCR5 entry inhibitors can have variable effects on other phenotypic features of Env [173]. 

Mutations outside V3 also modulate the effect of the primary resistance mutations in V3. For instance, a clone from a patient failing APL therapy contained eight mutations throughout gp41 and eleven mutations on the outer face of gp120 close to gp41 interacting residues. In this case, both gp120 and gp41 residues were required for full resistance [182]. Likewise, mutations in the V4 region can enhance resistance conferred by the V3 mutations, while other examples also indicate that V3 mutations might not be enough for full resistance [133,151,154,155,180,183]. The importance of Env context is also highlighted by experiments showing that transplanting resistance mutations or V3 regions into Envs of different strains often does not recapitulate the resistance phenotype, although in some cases it can [133,153,170,182,184]. For example, the FP mutations, described above, were unable to confer resistance when transferred to the JR_FL_ clone [172]. Thus, not only are different V3 resistance mutations required for different viruses, due to the slightly different interactions they make with the coreceptor, but mutations elsewhere in Env also indirectly affect coreceptor engagement or downstream conformational changes. Together, the variable sequences and long-range interactions in Env confound attempts to identify genetic signatures for CCR5 inhibitor resistance.

#### 3.3.3. Resistance Mechanisms

Despite the varied combinations of mutations that confer resistance, mechanisms of escape generally fall into two categories [186,187]. The first is a competitive mechanism, in which the resistance mutations enhance the efficiency of coreceptor use in the presence of the drug, so that the virus can still utilize the fewer number of drug-free coreceptors (Table 1, mechanism C (II)). The second is a non-competitive mechanism, in which the resistance mutations allow Env to use the drug‑bound conformation of the coreceptor (Table 1, mechanism B (II)). The competitive pathway was implicated in an *in vitro* AD101 selection study, in which resistant viruses were found to infect cells expressing lower amounts of CCR5 more efficiently [149]. Mutations that increase the binding affinity would provide a competitive advantage to the virus. However, in this study, this advantage was only seen early when resistance was still low, and later resistant viruses were no more efficient at using CCR5 than early viruses despite being nearly 20,000-fold more resistant [149]. 

Most often, escape involves a non-competitive mechanism of resistance, and many examples have been explored in detail [187]. Such resistant Envs from both *in vitro* and *in vivo* studies utilize the drug-bound form of CCR5. Operationally, this type of resistance has been detected in dose-response infectivity assays by finding a reduction in the plateau of maximal inhibition [129,133,151,155,171,188]. This plateau is limited by how efficiently the virus can use the drug-bound form of the coreceptor, as well as by the number of receptors available in a particular conformation on that target cell [151,153,171,172,182,185,189,190]. These issues are especially important given the variable conformational states and numbers of chemokine receptors in different cell types [111,191,192]. The utilization of the drug-bound form of CCR5 was confirmed in experiments demonstrating that a resistant virus was less sensitive to a different CCR5 entry inhibitor in the presence of saturating amount of the original entry inhibitor [151,171]. In those cases, the original inhibitor saturated CCR5 binding sites and obstructed the binding of the second inhibitor, demonstrating that the resistant virus utilized the drug bound form to gain entry through CCR5. 

A typical way that a resistant Env can use the drug-bound conformation of CCR5 is to depend more on the N-terminus of CCR5, rather than the ECL2, especially in the presence of an inhibitor that may disrupt interactions with the ECL2 [133,173,174,176,178,181,182]. The degree of resistance often mirrors the magnitude of altered N-terminal use [176]. However, different resistant Envs can vary in their dependence on certain regions within the N-terminus of CCR5. The FP mutant, for example, was less dependent on the CCR5 N-terminus and instead slightly more dependent on ECL2 [173]. Indeed, increased dependence for select ECL2 residues has been demonstrated for other clinical isolates as well [133,181,182]. Modeling has also provided insight into how various V3 mutations might create salt bridges and alter electrostatic surface potentials and other hydrogen-bond rearrangements to facilitate different V3 interactions with CCR5 [173,175,176,181]. Overall, how Env interacts with these critical CCR5 domains determines the ways it can alter its use of CCR5 to escape inhibition and possibly whether the resistant viruses have narrower or broader cross resistance profiles [188]. 

The ways that non-V3 mutations and Env context can influence coreceptor use is less clear, but almost certainly reflect critical interactions between residues from different Env domains that coordinate conformational changes to balance receptor engagement with the propensity of gp41 to refold into the 6HB. Altering coreceptor use to accommodate an inhibitor must avoid detrimental changes in interactions between gp120 and gp41 that could promote premature triggering and inactivation of Env. Data support the concept that the V3 domain, which is predicted to be at the center of the trimer interface, is an allosteric rheostat that reduces the energy of activation for fusion‑inducing, conformational changes in Env [193]. Such control may be needed for clamping down on gp41 to prevent inappropriate triggering to its more stable conformation. On the other hand, reducing the allosteric hold on gp41 may be necessary if the drug-bound conformation of CCR5 is used less efficiently. This allosteric hold may affect entry kinetics, which has been shown to affect sensitivity to CCR5 entry inhibitors [194]. Furthermore, recent data indicate that mutations in VCV‑resistant viruses restore entry kinetics to wild-type levels in the presence of the inhibitor [195]. Altogether, it remains possible that these gp41 changes alter gp120-gp41 interactions to allow more efficient use of different conformations of CCR5 through cooperative movements across the Env [172,185,196]. 

Finally, the genetic pathways selected for resistance must take overall fitness into account. While resistant mutants selected in the presence of AD101 were not associated with a fitness loss, there is evidence that VCV resistance reduced fitness [163,197]. Compensatory mutations frequently increased infectivity, and Env strain affected resistance phenotypes [173,176]. Deep sequencing of VCV-treated patients showed rapid expansion of V3 sequence diversity and minor variants during treatment, indicating that variants that are initially less than 1% of the quasispecies can be clinically relevant [198]. Furthermore, co-evolution between gp120 and gp41 may help to maximize fitness and entry kinetics [199]. The quasispecies, therefore, provides a ready source of variants for sampling and finding an efficient pathway for resistance. The dominant genetic pathways would be determined by both resistance and fitness considerations. 

### 3.4. Summary

Resistance mutations to entry inhibitors that target Env interactions with the coreceptor emerge principally within the V3 domain, which is a critical determinant for coreceptor binding. However, as seen with resistance to entry inhibitors that target gp120 interactions with CD4, coreceptor inhibitor resistance mutations were often found throughout Env. Other determinants outside the coreceptor binding site modulate the magnitude of resistance, and in some cases fully account for it (e.g., FP mutations). Furthermore, the effects of the mutations on the resistance phenotype depend on the Env strain. The lack of a resistance signature sequence reflects the complex network of interactions within Env, including variable loops, which work together for coreceptor engagement that ensures proper triggering of gp41 and fusion pore formation. 

The mechanisms underlying the varied genetic pathways for resistance are more limited. Although a competitive mechanism in which resistance mutations improve binding to the inhibitor-free form of the coreceptor has been described in an example of moderate resistance (Table 1 mechanism C (II)), resistance mutations in Env typically permit escape through a non-competitive mechanism. In this latter scenario, Env adjusts to bind to an altered conformation of the coreceptor imposed by the bound inhibitor, so that even saturating levels of bound inhibitor cannot block virus entry (Table 1, mechanism B (II)). Typically, this results in a plateau of maximal inhibition in infectivity assays that reflects the efficiency of virus entry using the inhibitor-bound conformation of the coreceptor. 

Selection for viruses that use an alternate coreceptor not targeted by the inhibitor occurs primarily in the clinical setting of CCR5 inhibitor therapy (Table 1, mechanism C (II)). A robust quasispecies and the availability of diverse, permissible cell types in a variety of tissues provide many possibilities for selecting viral variants that can use CXCR4 and bypass CCR5 inhibition entirely. True coreceptor switching through the accumulation of mutations within Env apparently presents a higher genetic barrier and/or fitness cost than other resistance mechanisms. Finally, despite the high frequency of detecting X4 virus outgrowths in subjects failing CCR5 inhibitor therapy, R5 viruses are detected during virologic failure in some patients. Resistance in R5 viruses might also occur more often in subjects infected with certain HIV subtypes. In these cases, resistant viruses that can use inhibitor‑bound conformations are likely present. 

## 4. gp41 Fusion Inhibitors

### 4.1. Introduction

The next class of entry inhibitors targets the fusion activity of gp41. After gp120 engages receptors, a series of still poorly defined refolding events frees the hydrophobic fusion peptide at the N terminus of gp41 so that it can insert into the target cell membrane (forming the prehairpin intermediate) [98]. While bridging both viral and cellular membranes, the two heptad-repeat regions in the gp41 ectodomain (HR1 and HR2) self-assemble to form a compact, thermostable six-helix bundle (6HB) structure (also referred to as trimer-of-hairpins conformation) [25,200]. Concurrently, the energy of refolding drives membrane merger between the virus and target cell and stabilizes formation of an expanding fusion pore that eventually allows the viral core to pass into the cytoplasm [24,201]. 

**Figure 3:**
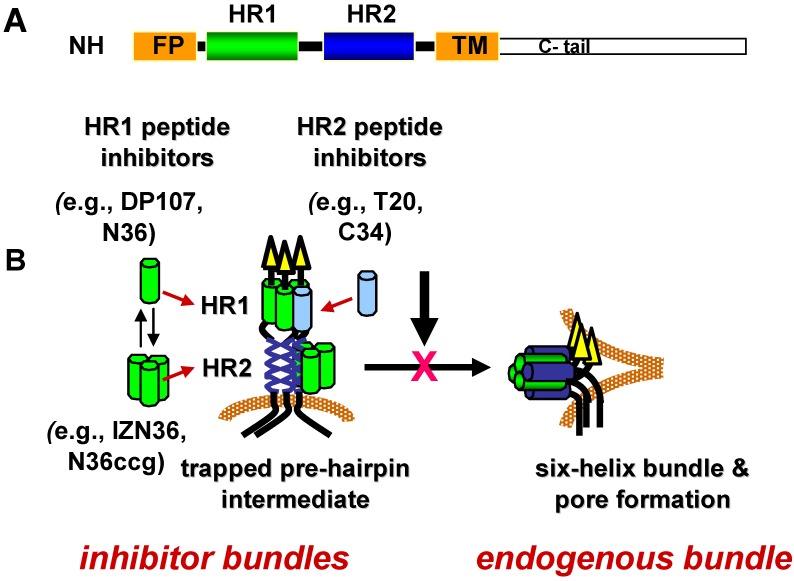
gp41 heptad-repeat domains and fusion inhibitors. (**A**) Schematic diagram of gp41 domains. NH2 indicates N-terminus. FP indicates fusion peptide. HR1 is the N‑terminal heptad repeat. HR2 is the C-terminal heptad repeat. TM indicates transmembrane domain. C-tail indicates the cytoplasmic domain. (**B**) Model of dominant negative inhibition by heptad-repeat peptides. HR1 and HR2 peptides mimic the HR1 and HR2 segments, respectively, in the gp41 ectodomain. During receptor-induced conformational changes in the envelope glycoprotein, the HR1 and HR2 become more exposed as the fusion peptide (FP) inserts into the host cell membrane. This conformation is referred to as the pre-hairpin fusion intermediate. Before the HR1 and HR2 continue to self assemble to form the six-helix bundle (endogenous bundle), the HR1 or HR2 peptides gain access to the endogenous HR1 or HR2 to form a peptide-gp41 six-helix bundle (inhibitor bundle). In this dominant negative mechanism of inhibition, the peptide inhibitor interrupts formation of the endogenous bundle and pore formation needed for promoting membrane fusion between the virus and host cell membrane.

Specific conformations in the refolding process have temporal windows during which they are vulnerable to inhibition. The duration of vulnerability is likely limited by the kinetics of refolding. As discussed in more detail in the sections below, lead inhibitors in this class are peptides corresponding to residues in HR1 and HR2 (Figure 3A) that appear to inhibit Env after receptor activation and before formation of the gp41 6HB in a dominant negative manner [202,203,204]. According to this mechanism of inhibition, the HR peptide inhibitors bind to a fusion-intermediate conformation of gp41 to form peptide-gp41 6HB-like structure (inhibitor bundle) (Figure 3B). In this way, the peptides trap gp41 during conformational changes and prevent gp41 from forming a 6HB (endogenous bundle) that can participate in fusion. 

Using available structures of the 6HB and thermodynamic considerations, various peptide inhibitors have been designed for improved potency, but to date, only one inhibitor, enfuvirtide (also known as T20 or DP178), has been approved for use in the clinic [205]. As a peptide, enfuvirtide must be administered by injection, making it a less attractive therapeutic option compared to the many orally‑available antiretroviral drugs. Still, new types of fusion inhibitors targeting gp41, including small molecules, are under development, and they can be used as additional tools for deciphering how gp120 and gp41 coordinate conformational changes to mediate virus entry [10].

### 4.2. HR2 Fusion Inhibitors

#### 4.2.1. T20

The approval of enfuvirtide, a peptide corresponding to gp160 residues 638–673 (gp41 residues 127–162) in the HR2 of the LAI strain, broke new ground in 2003 as the first entry inhibitor available for treating HIV infection [206,207,208]. Its discovery as a fusion inhibitor in the early 1990’s [209], which was slightly predated by the identification of inhibitory properties of another peptide corresponding to HR1 (DP107) [210], was an unexpected finding in experiments aimed at investigating the function of potential helical regions in Env. At about the same time, a different but overlapping HR2 peptide (SJ-2176) corresponding to gp160 residues 630-659, with potent *in vitro* inhibitory properties was also discovered independently by another group studying domain interactions in Env [211]. 

Although the mechanism of inhibition of these HR peptides was initially enigmatic, the elucidation of high-resolution structures of the gp41 core domain offered an explanation; the peptides mimic gp41 (endogenous) HR1 and HR2 and consequently interfere with formation of the gp41 (endogenous) 6HB (Figure 3B) [212,213,214,215,216,217,218]. The main inhibitory activity of enfurvirtude and its derivatives, as well as other overlapping peptides, has been attributed to the ability of the peptides to bind to the hydrophobic grooves of the HR1 coiled-coil core of the 6HB, though additional inhibitory activities have been reported for some HR2 peptides [219,220,221,222]. That the peptides can effectively prevail over the endogenous HR in forming a bundle also indicates that self assembly of the endogenous HR1 and HR2 are restricted during refolding, presumably by other regions of Env. Studies showing that HR peptides preferentially bind to gp41 after receptor activation further point to a transient fusion-intermediate conformation as the target of HR peptide inhibitors [223,224,225,226]. 

**Figure 4:**
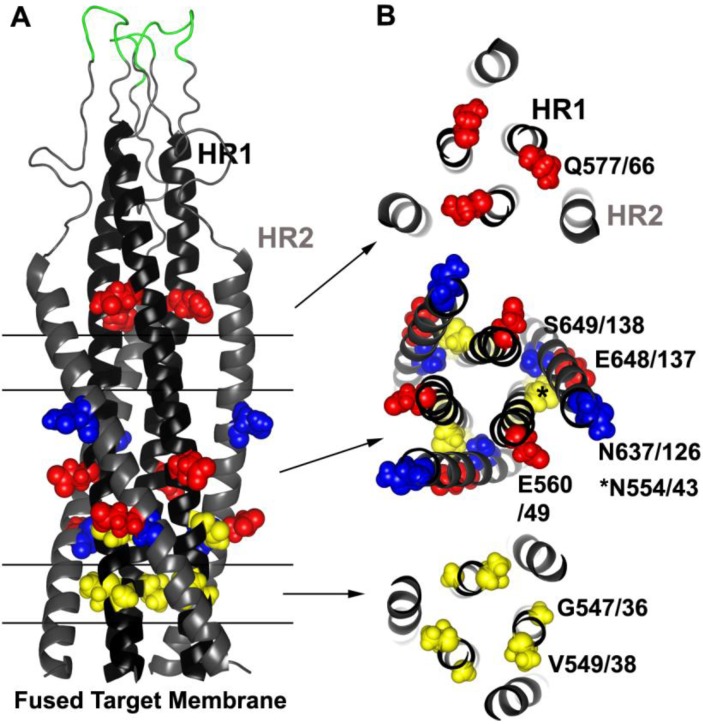
Common resistance mutations selected by HR1 and HR2 peptide inhibitors. (**A**) Side view model of the 6HB derived from the SIV gp41-ectodomain NMR structure [250,251] (Protein Data Bank entry 1IF3). Parallel lines and arrows indicate the relevant cross sections in (**B**). T20 and other early-generation HR2 peptides tend to select for residues in the HR1. Sites of three common resistance mutations occurring in HR1 (G547/36, V549/38 and N554/43, gp160/gp41 numbering according to the HXB2 clone) are highlighted in yellow spheres. Two compensatory mutations in HR2 that frequently appear at two locations (N637/126 and S649/138) are indicted by blue spheres. HR1‑peptide inhibitors, which may target HR2 and/or HR1, commonly select for mutations at three positions (Q577/66, E560/49 and E648/137) and are indicated by red spheres. Residues adjacent to the disulfide loop (green) can also confer resistance to certain small molecule inhibitors. Residues in yellow tend to destabilize the bundle, and residues in red and blue tend to stabilize the bundle.

Resistance studies involving T20 confirm its binding site on gp41 (Figure 4). One of the first *in vitro* resistance studies showed that at least two HR1 mutations, one each in gp160 positions 547 and 549 (gp41 position 36 and 38, respectively), involving G547D (gp41 G36D) and V549I/M (gp41 V38I/M), were needed to confer resistance to replicating virus [227]. Indeed, numerous other *in vitro* and clinical studies have confirmed that mutations within the so-called GIV motif (gp160 residues 547–549 or gp41 residues 36–38), as well as other mutations along HR1 are the primary hot spot for T20 resistance [228,229,230,231,232,233,234,235,236,237,238,239,240,241,242]. These mutations emerge quickly under selection and are seen in many HIV subtypes. Residues in these positions are also extremely conserved, and these resistance mutations are rarely observed in untreated patients [229,243,244,245,246,247,248,249]. 

Often pairs of mutations within HR1, sometimes in combination with HR2 mutations, are needed to increase resistance, and they tend to accumulate over the course of treatment [227,229,233,234,235,239,241,249,252]. In most cases these HR2 mutations, such as S649A (gp41 S138A), N637K (gp41 N126K), and E648K (gp41 E137K), provide little if any resistance on their own unless paired with HR1 mutations, and the HR2 mutations may help to increase virus fitness [231,233,234,235,239,253,254,255]. However, sometimes there are specific patterns to the mutations. Often no more than a pair of mutations occur within residues 547–556 (gp41 residues 36–45), and mutations such as N554D (gp41 N43D) are usually not observed together with changes at gp41 position 547 (gp41 36) or 549 (gp41 38), despite the high frequency of mutations in the GIV region in resistant Envs [237,256]. 

In contrast to resistance mutations selected by chemokine receptor inhibitors, mutations selected by fusion inhibitors often confer resistance regardless of the Env context, and therefore can be used as *bona fide* resistance signature sequences. However, Env context still matters, as was initially observed by the wide susceptibility differences of various Envs to T20, which may even constrain the development of resistance [229,241,244,257,258,259]. Additionally, placing the double mutations of N553T and N554S (gp41 N42T and N43S) into two different Env contexts can result in more than a two log difference in resistance between the different Envs [241]. Furthermore, resistance mutations may not simply accumulate over time. Rather, their emergence can be dynamic and change over the course of treatment within a patient [231,233,240]. In two studies, the first resistance mutations that dominated early time points occurred within the GIV region, but at later time points these mutations were not seen, and the N554D (gp41 N43D) mutation dominated [233,237]. 

The resistance mutations on HR1, where T20 docks onto the coiled-coil core, destabilized 6HB interactions, suggesting that the resistance mutations lower the affinity of the inhibitor for HR1 [227,232,241,242] (Table 1, mechanism A (III)). Although efficient at impairing T20 binding to gp41, these destabilizing mutations could also reduce virus fitness because 6HB interactions between the mutated endogenous HR1 and the endogenous HR2 would also be negatively affected [233,258,260,261]. In many cases, especially in the clinical setting, HR2 mutations appear later during treatment to compensate for the destabilizing HR1 mutations. Using peptide models and crystallographic data, it was shown that these HR2 mutations can enhance the thermodynamic stability of a 6HB, in some cases through direct interactions with the mutated residue [232,235,255,262]. In another interesting example of Env remodeling, resistance evolved to depend on the presence of T20, which was presumably needed to temporarily bind to Env during a critical stage of refolding [231,263] (Table 1, mechanism B (III)). In general, these compensatory pathways maintain structural stability, which enhance virus fitness without losing the ability to escape T20 inhibition. 

#### 4.2.2. Next-Generation HR2 Peptides

Based on information from T20 resistance studies, next-generation HR2 peptide inhibitors were designed to enhance interactions with HR1 that could overcome common T20 resistance mutations [8,10]. Notably, it had already been observed that shifting the sequence and number of amino acids in HR2 peptides can affect potency [209,211,216]. One such peptide, C34, an HR2 peptide shifted N‑terminal to T20 (gp160 residues 628–661), has additional interactions with a hydrophobic pocket in HR1 and is generally more potent than T20 [218,264,265]. Next-generation HR2 peptides that are more potent than T20 and active against some T20-resistant viruses include T649 (which differs from C34 by two amino acids), CP621–652, (further shifted to the N-terminus of C34) and T1249, which contains an optimized HR2 39-mer utilizing HIV-1, HIV-2 and SIV sequences [227,266,267,268,269]. The two additional residues (M626 and T627) included in the CP621–652 peptide make a hook-like structure that helps to stabilize 6HB interactions and enhance the peptide’s potency against HIV, including T20‑resistant viruses [270,271]. However, *in vitro* resistance studies involving C34 and T1249, as well as clinical studies involving T1249, indicate that resistance genotypes are still remarkably similar to those obtained with T20 [232,272,273,274,275,276]. One notable exception is that position 549 (gp41 position 38) tends to be mutated to a charged residue, rather than the more typical alanine or methionine residue observed with T20 resistance [269,274,276]. Given the higher potential binding energy of the newer generation HR2 peptides to the endogenous HR1, mutation of residue 549 (gp41 residue 38) to a charged residue may be needed to further destabilize interactions through charge repulsion between residue 549 (gp41 residue 38) and the glutamic acid at position 657 (gp41 position 146) in HR2 [276,277]. Mutations that cause electrostatic repulsion, reduced contact, steric obstruction, or electrostatic attraction between residues in the 6HB have been described as types of mechanisms that confer resistance to T20 [277]. 

Other strategies were followed to make HR2 peptides more soluble and helical. For instance, modifying a series of solvent-accessible sites with glutamate and lysine improved the thermodynamic properties of the peptides sifuviritide, CP32M, SC34, and SC34EK and their potency against T20‑resistant Envs [265,278,279,280,281]. Furthermore, crystal structures of some of these new inhibitors show novel structural features that help to explain their increased potency and how these modifications enhance inhibitor-target interactions [282,283]. Despite improvements, single mutations on HR1 could still confer moderate resistance, but much less resistance than earlier generation inhibitors [280,284,285]. Additionally, multiple mutations in HR1 and HR2 were often needed to maximize resistance, especially for SC34 and SC34EK peptides, where single mutations conferred little if any resistance [280,285,286]. Interestingly, the SC peptides, which differ by a charge polarity, show diminished cross resistance to each other and other fusion peptide inhibitors, underscoring how modified HR2 peptides can have distinct resistance profiles [286]. Further modification of these peptides to include HR2 resistance mutations, a strategy previously demonstrated to enhance the affinity between HR2 and its target, was also able to improve potency for SC-derived resistant mutants [286,287]. 

Despite strategies to improve inhibitors by including residues that would increase the thermodynamic stability of the bundle formed with the endogenous HR1, there are limits for how potent a given peptide can be based solely on its affinity for HR1 [288,289,290]. In fact, the third generation peptide inhibitor, T2635, has enhanced helicity and forms a much more stable inhibitor bundle with the HR1 compared to T1249 and T20, yet it displays little difference in potency against the IIIB isolate [288]. Nonetheless, viruses resistant to T20 were again more sensitive (up to >3,600× compared to T20) to T2635 [288]. 

The favorable anti-viral properties of T2635 appear to translate to a higher genetic barrier to resistance. An *in vitro* resistance study demonstrated that resistance to T2635 was much harder to acquire than to T20 or T1249, and the profile of resistance mutations was much different than is typically observed for HR2 peptides [291]. In particular, multiple mutations were spread out across the gp41 ectodomain. They included mutations in the fusion peptide, HR1, the loop between HR1 and HR2, HR2, and the membrane proximal external region (MPER) region. Mutations were also noted in gp120 that may compensate for the reduced infectivity resulting from the gp41 resistance mutations. 

In an effort to overcome the intrinsic limits to HR2-peptide affinity, some groups have tried to anchor the HR2 peptide to the membrane by attaching an octyl group, a cholesterol moiety, or a membrane-spanning domain (MSD) [292,293,294,295]. These inhibitors have improved potency relative to the unmodified peptides, and in the case of the cholesterol-anchored C34 peptide, potency reached low picomolar levels. Despite these efforts, this class of inhibitors, at least the MSD-conjugated inhibitors for which resistance studies are available, can still select for resistant viruses [296,297]. However, for the optimized MSD-conjugated inhibitor, resistance did not develop rapidly, and it selected for an Env containing multiple cooperating mutations in both gp120 and gp41 [296,297]. Similarly, escape from a novel peptide scaffold based on D-stereochemistry was also difficult to achieve, apparently because its high affinity for HR1 created a built in capacitance for enduring the resistance mutations [298]. In all, the high genetic barrier to resistance suggests that the resistance mechanism used by the latest generation of entry inhibitors differs from that used by T20 and does not appear to simply depend on mutating contact residues that directly impair inhibitor binding.

The notion that regions not directly involved in inhibitor binding affect susceptibility to inhibition by HR2 peptides is supported by reports showing that baseline sensitivity to T20 and other HR2 inhibitors varies over a large range, even though the HR target is highly conserved [229,244,257]. In part, determinants for this sensitivity can lie outside of the HR1 resistant mutation hot spots and have been shown to lie within the V3 and adjacent regions thought to affect coreceptor binding [194,244,266,299,300]. Accordingly, a correlation between coreceptor affinity, entry kinetics, and T20 susceptibility was reported [194]. These experiments were built on the knowledge that an intermediate conformation in the fusion process, between CD4 binding and 6HB formation, is transient and presents a limited opportunity for T20 to bind to gp41 and inhibit 6HB formation. Consistent with these data, a model was proposed that high affinity coreceptor binding allows Env to progress through the fusion process more rapidly, consequently shortening the lifetime of the fusion-intermediate conformation and reducing the sensitivity to T20 [194]. Therefore, sequences in Env that modulate coreceptor affinity or enhance transitioning through susceptible intermediates may also affect T20 sensitivity. 

Experiments measuring fusion kinetics of resistant Envs derived *in vivo*, however, indicate that the resistance mutations can actually slow down overall fusion kinetics [254,301]. These data are consistent with work demonstrating that HR1 mutations reduce replication kinetics and can lower infectivity [260,291]. Compensatory HR2 mutations can nevertheless restore fusion kinetics back to, but not faster than, wildtype rates [254]. Therefore, the genetic pathway to enhance fusion kinetics beyond the rate of the sensitive parental Env may be difficult to achieve and limited by competing fitness considerations. Combining mutations in HR1 and HR2 to modulate 6HB stability and restore entry kinetics appears to be the preferred resistance pathway. 

Overall fusion kinetics, however, may not be the only kinetic parameter that can inform susceptibility to entry inhibitors. Recently it was shown that the lifetime of an early fusion intermediate, rather than the lifetime of HR2 susceptibility, correlated better to potency of HR2 peptides [302]. Among different Envs, differences in the lifetimes of discrete intermediates that are susceptible to the inhibitors may not be evident from measurements of overall fusion kinetics. Additionally, the HR1 and HR2 regions may become accessible at different time points along the fusion pathway [289,303]. The more complex resistance profiles selected by newer generation fusion inhibitors may indicate that Env needs to restructure itself to gain a kinetic advantage over the inhibitor. However, many issues still need to be resolved to fully understand the relationship between fusion kinetics and resistance to HR peptides as many parameters affect fusion kinetics, including Env strain, receptor levels, target cells, and cell-cell *versus* virus-cell fusion. 

### 4.3. HR1 Peptide Inhibitors

The first HR1 inhibitory peptide DP107, corresponding to gp160 residues 558–595 from the LAI strain (gp41 residues 47–84), prevented HIV infection when used at micromolar concentrations, and its potency correlated with its ability to form a coiled-coil structure [210,304]. In solution, HR1 peptides can exist as monomers, dimers, tetramers, and aggregates [216,305,306,307,308]. Modifications that stabilize trimers and reduce aggregation improve potency, but HR1 peptide inhibitors have not yet reached the clinic [10]. Examples of modifications that improve HR1 potency include, but are not limited to, stabilized trimers [309,310,311,312,313], lipohilic tails [314,315], or coupling two HR2 domains in tandem with 3 HR1 domains to form a five-helix bundle (5HB) that exposes one groove of the HR1 coiled-coil core [316]. The dominant negative mode of inhibition predicts that HR1 peptides, in contrast to HR2 peptides, could potentially interact with two sites in gp41 (Figure 3B). In one case, HR1 peptides, most likely as a trimer, could bind to the endogenous HR2 to form a peptide-gp41 6HB (inhibitor bundle). Alternatively, HR1 peptides, perhaps as monomers or dimers, could intercalate into the endogenous HR1 coiled coil and subsequently interfere with formation of the gp41 6HB (endogeous bundle) that can mediate fusion [317]. 

Research on HR1 peptides has lagged in comparison to HR2 peptides, but recent work with several HR1 peptides has begun to identify new resistance pathways and insights into the fusion process. Using the JR_CSF_ clone, resistance to the HR1 peptides N44 and N36 (gp160 residues 546–590 and 546–581 of the HXB2 clone, respectively) selected for two genetic pathways characterized by Envs containing either, but not limited to, a E560K mutation in HR1 or E648K mutation in HR2 [318,319,320] (Figure 4). By contrast, the IZN36 inhibitor, comprised of N36 fused to the synthetic IZ trimerization domain [309], selected predominantly for the HR1 E560K pathway [320]. 

In contrast to mutations leading to HR2 peptide resistance, these HR1 resistance mutations enhanced the thermodynamic stability of the 6HB. Furthermore, a second mutation, Q577R, which was identified in some resistance cultures from both pathways, significantly increased both bundle stability and resistance (Figure 4). N36 resistance studies using clones from a different strain (NL4-3) also recovered the E648K mutation that similarly increased stability of the 6HB in that strain [321]. Surprisingly, cross resistance to T20, albeit at reduced levels (~2–20-fold), was also observed in each of these studies, suggesting that increasing bundle stability can provide the virus with a general advantage against inhibitors targeting gp41 fusion intermediates (Table 1, mechanism C (III)). Interestingly, these three resistance mutations have been identified in some resistance screens using HR2-like peptides, underscoring the contribution of the E560K, E648K, and Q577R mutations to the overall resistance phenotype to peptide fusion inhibitors [234,239,253,255,291,298]. 

Selection for mutations that increase 6HB stability might seem like a paradoxical solution for escaping inhibition by HR peptides, because mutations that increase stability of the endogenous bundle could also increase the affinity of the HR inhibitor for its target site on Env (Figure 3B). For instance, the HR2 E648K mutation would not only improve interactions with endogenous HR1 (forming the endogenous bundle), but it could also improve interactions with the HR1 peptide inhibitor (forming the inhibitor bundle). The E560K mutation, on the other hand, might be less advantageous to the HR1 inhibitor if it targets the endogenous HR2. However, the E560K mutation could improve interactions of the inhibitor with the endogenous HR1 coiled coil. Because the mutations confer cross resistance to multiple HR peptide inhibitors, they do not provide unambiguous information on the target sites for the HR1 peptides. Conceivably, HR1 peptides, which are in a dynamic equilibrium between monomers and oligomers, might be able to bind to both HR1 and HR2, respectively. If this were the case, a more general escape mechanism that confers cross resistance to all forms of the inhibitor might be needed. 

How mutations that increase bundle stability preferentially benefit the virus and confer resistance to multiple HR peptide inhibitors could be explained in two ways. Given the temporal constraints in the fusion process, it seems likely that these mutations might favor the resistant virus if they increase the rate of formation of the endogenous bundle more than the inhibitor bundle. In fact, it has been demonstrated that once the affinity of HR peptides or the 5HB inhibitor reaches a threshold level, the rate of association, rather than target affinity, correlates better with *in vitro* potency [289,322]. 

Alternatively, the resistance mutations could provide a thermodynamic advantage to the endogenous bundle over the inhibitor bundle by increasing the affinity of the interactions in the endogenous bundle more than the inhibitor bundle. Nonetheless, this mechanism may be limited by the off rate of the inhibitor or endogenous HR forming the helix bundle, as well as by the relative lifetimes of these intermediates [289]. In either case, resistance mutations arising in the HR domain that correspond to the inhibitor binding site would be expected to give the resistant virus an advantage, because the inhibitor bundle would lack such mutations in the helices contributed by the inhibitor. More work is needed to clarify the complex issues of where and when the HR inhibitors bind to specific conformations of Env, and how long the window for inhibition lasts. Such studies with HR inhibitors aid our understanding of HIV entry. 

**Table 1:** Scheme of potential resistance mechanisms for different classes of entry inhibitors. For inhibitors that interfere with gp120 interactions with the CD4 receptor, resistance mutations in or near the receptor binding site commonly emerge. These mutations may prevent inhibitor binding to its target (mechanism A (I)) or allow gp120 to bind to the receptor and mediate entry while the inhibitor remains bound to its target (mechanism B (I)), or improve gp120 affinity for CD4 so that gp120 outcompetes the inhibitor for its target (mechanism C (I)). For inhibitors that interfere with gp120 interactions with the chemokine coreceptor, the patterns of resistance mutations are more complex and widely distributed across the envelope glycoprotein, though many emerge in the V3 region. The mechanisms of resistance for chemokine receptor inhibitors typically involves use of the coreceptor in the presence of bound inhibitor (mechanism B (II)) or selection for viruses that use a different chemokine receptor or possibly use the coreceptor with higher efficiency to outcompete the inhibitor (mechanism B(III)). For inhibitors that interrupt gp41 six-helix bundle (6HB) formation, primary resistance mutations emerge in the heptad repeat regions. Peptides corresponding to HR2 sequences and targeting the HR1 in gp41 to form an inhibitor bundle usually select for mutations in HR1 that directly interfere with inhibitor binding to that region and sometimes contain compensatory mutations in HR2 that can restore bundle stability (mechanism A (III) or, may allow (transient) binding of the inhibitor to the HR1 so as to facilitate gp41 refolding and function (mechanism B (III)). Peptides containing HR1 sequences also select for primary resistance mutation in both HR1 and HR2 regions, but instead use a mechanism that involves improved stability of the 6HB, which may increase the rate of formation of the 6HB (mechanism C (III)). These mutations also often confer broader cross resistance to a variety of HR1 and HR2 peptide inhibitors, compared to the resistance mutations that emerge under selection by HR2 peptides. These schemes do not attempt to take into account all possibilities and mechanistic categories may overlap in some cases. X = inhibitor binding to the envelope glycoprotein, CD4 receptor, or chemokine coreceptor. gp41 heptad repeat 1 (HR1) is green. gp41 heptad repeat 2 (HR2) is dark blue. HR2 peptide inhibitor is light blue. HR1 peptide inhibitor is green and present in an equilibrium of monomers-oligomers. Small colored shapes within figures indicate mutations. +/− indicates potential other mutations that influence resistance levels. *n.a.* = non-applicable.

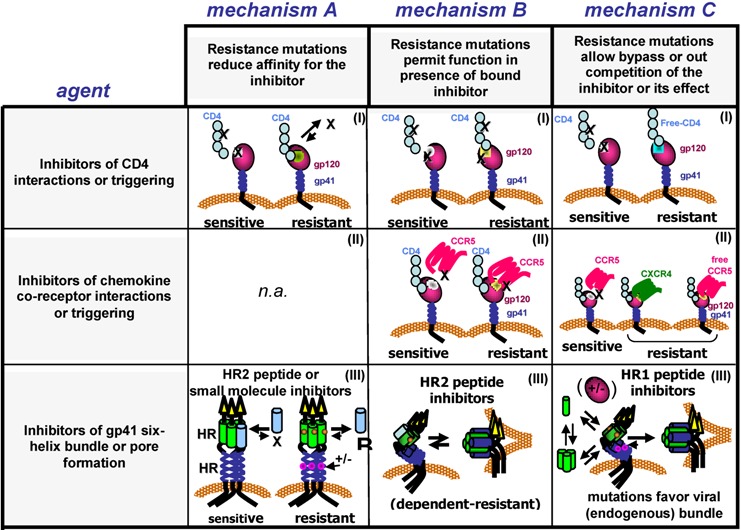

### 4.4. Summary

Peptides corresponding to the HR1 and HR2 regions of gp41 can be potent inhibitors of HIV infection. They appear to inhibit HIV by replacing, at least transiently, the corresponding endogenous HR segments in gp41 to interrupt formation of the endogenous 6HB during membrane fusion (Figure 3B). Because the peptides mimic native interactions in Env and inhibit in a dominant negative manner, the solutions for the virus to escape inhibition are restricted by the need for gp41 to form an endogenous 6HB that can participate in the fusion process. HR2 inhibitors tend to select for Envs that contain mutations in HR1 that destabilize the 6HB, followed by compensatory mutations in HR2 that restore fusion kinetics and/or bundle stability (Table 1, mechanism A (III)). By contrast, HR1 peptides select for mutations in HR1 and HR2 that enhance 6HB stability in way that preferentially favors the resistant virus, which may relate to enhanced kinetics of 6HB folding (Table 1, mechanism C (III)). 

As more potent, newer generation peptide inhibitors are being developed, their resistance profiles are becoming more complex, both in terms of the number of mutations and mechanism of resistance. These changes are constrained by the relative fitness of the resistant virus. While HR1 and HR2 mutations dominate resistance genotypes of these newer generation peptide inhibitors, compensatory mutations throughout the envelope, including in gp120, are often needed and are seen especially in Envs from patients treated for a long time. Understanding the relationship between fitness and resistance will be an important step in understanding how HIV acquires resistance to gp41 inhibitors. 

## 5. Other Entry Inhibitors

Several entry inhibitors have binding sites and mechanisms of action that are not clearly defined and therefore do not fit into one of the inhibitor classes described in the previous sections. However, their resistance patterns still provide valuable insights into HIV entry. For instance, studies of resistance to a diverse set of inhibitors that were found some time ago, including the oligocationic compound ALX40-4C, an amphotericin B derivative MS8209, and the oligonucleotide AR177 (T30177, Zintevir), all depend on mutations in gp120, especially within the V3 domain [323,324,325]. Another peptide called Virus-Inhibitory Peptide (VIRIP), a fragment of alpha-1-antitrypsin found in human hemofiltrate, can inhibit a wide range of HIV-1 strains independent of binding to CD4 or CCR5 receptors [326]. Originally VIRIP and its more potent derivatives were thought to bind to the fusion peptide (FP) region on gp41 [326]. Although selection with VIRIP did not yield resistance in this study, exchanging the FP region of the NL4-3 clone with the FP region of the SIVmac239 clone did reduce susceptibility to VIRIP, and, interestingly, increased sensitivity to T20. In a later study, viruses resistant to VIRIP had no mutations in the FP region, but instead had an accumulation of mutations in both gp120 and gp41 that was necessary for maximum resistance [327,328]. 

Another inhibitor called RPR103611, a triterpene derived of betulinic acid, is specific for only certain Env strains and inhibits a post-CD4 binding step [329]. Sensitivity determinants for RPR103611 were conferred by gp41, which included point mutations within and adjacent to the disulfide loop (DSL) between HR1 and HR2 [330,331]. Interestingly, resistance seemed to correlate with Envs that have more stable gp120-gp41 interactions [330]. 

By contrast, a stereoisomer of RPR103611 called IC9564 has broader inhibitory activity and selected a different set of resistance mutations. Resistance mutations were found in three regions: G237R and R252K mutations were found in the inner domain of gp120, and a single mutation was found in both the bridging sheet and V3. Each of these mutations individually was sufficient for conferring resistance to IC9564 [332,333,334]. Some of these mutations have been shown to reduce exposure of neutralizing antibody epitopes in the presence or absence of IC9564, indicating that the resistant Envs may alter Env conformational transitions [333,335]. Based on modeling and competition experiments with the V3 mAb, the binding site for IC9564 is thought to be within the V3 domain, contrasting the original hypothesis that RPR103611 binds to gp41 [334].

Recently the benzene sulfonamide PF-68742, a small molecule inhibitor that is unique from the inhibitors described above, developed resistance mutations in locations similar to those found for VIRIP and RPR103611 [336]. Resistance mutations were identified in the FP region, as well as within and proximal to the DSL [336]. Curiously, when the G514R single point mutation was introduced into the FP, infectivity was dramatically reduced in the absence of the inhibitor, but partially restored in the presence of the inhibitor. This finding indicates that resistance does not require a block to inhibitor binding, but rather supports the proposal that the resistance mechanism to PF-68742 involves complex Env interactions that can be due in part to inhibitor binding [336] (similar to HR2 peptide inhibitors in Table 1, mechanism B (III). 

Although uncertainties remain about the binding sites and mechanisms of action of the inhibitors described in this section, the resistance phenotypes have frequently been ascribed to modulation of the gp41/gp120 interactive interface [327,328,330,331,332,333,335,336]. Notably, the DSL and adjacent region between HR1 and HR2 have been implicated in gp41-gp120 interactions and can influence gp160 cleavage just N terminal to the FP region [337,338,339,340,341,342,343,344,345]. The gp120 inner domain, although not in direct contact with CD4, participates in a network of interactions that can modulate CD4 activation and contact with gp41 [40,346]. The V3 region also contributes to the stability of critical gp120-gp41 interactions, which together with other inter-domain contacts, may help to control conformational transitions leading to membrane fusion [193,347,348,349,350,351]. The cumulative effect of these cooperative interactions from disparate regions of Env shapes its phenotype [350]. Furthermore, recent structural work on the trimer, although at low resolution, provides an opportunity to observe how the different gp120 structural domains interact across the trimer interface and how they contact gp41 [97,352]. The resistance mutations described in this section, like those described in other sections, may help to define these cooperative networks that link gp41 and gp120.

## 6. Final Perspective

Resistance studies involving all major classes of HIV entry inhibitors highlight the capacity of Env to restructure itself to escape inhibition. Resistance can occur through mutations that destabilize inhibitor binding to its target site on Env, which only occasionally highlight signature residues that can be used predict resistance to a particular inhibitor. More often, resistance involves the accumulation of multiple mutations across Env that together modify conformational transitions to enhance virus entry, sometimes even using the inhibitor. For all inhibitor classes, mutations in domains outside the inhibitor or receptor binding sites can frequently be important for resistance. For example, mutations in gp41 can contribute to resistance to CCR5 as well as CD4 inhibitors. Similarly, gp120 mutations can modulate the potency of inhibition by gp41 peptide fusion inhibitors. Importantly, such resistance mutations are often context dependent and are unable to confer resistance in an Env from another strain. 

The long-range, cooperative interactions among different regions of Env that control entry are not well understood. However, as described in Section 5 and throughout the resistance studies presented in this review, more attention is being directed at uncovering these complex networks. Resistance studies to different classes of entry inhibitors illustrate how variable regions in Env build in considerable flexibility for allowing Env to adjust to a changing environment while preserving the functions of its conserved core domains. This plasticity allows the virus to maintain a chronic infection in a host with evolving immune responses and dynamic changes in target cells. The complex quasispecies further provide a rich source for selecting fit and resistant viruses. Therefore, inhibitor design must take into account the limits that improvements in inhibitor affinity for its target can have on the development of resistance. Considerations should also be given to how resistance might develop so that extra features can be built into the inhibitors to make it more difficult for the virus to escape. 
